# IVUS-guided wiring using the deep vein as a landmark (IDEAL) technique for treating chronic total occlusions in peripheral artery disease

**DOI:** 10.1186/s42155-025-00584-5

**Published:** 2025-08-15

**Authors:** Yohei Ueno, Mitsuo Sobajima, Teruhiko Imamura, Hiroshi Onoda, Ryuichi Ushijima, Hiroshi Ueno, Koichiro Kinugawa

**Affiliations:** The Second Department of Internal Medicine, Graduate School of Medicine, University of Toyama2630 , Sugitani, Toyama, Japan

**Keywords:** Novel technique, Intravascular ultrasound, Deep vein, IVUS-guided wiring, Endovascular treatment, Peripheral artery disease, Chronic total occlusion

## Abstract

**Background:**

Endovascular therapy (EVT) is a well-established revascularization strategy for patients with peripheral artery disease. However, achieving optimal wire crossing in complex chronic total occlusion lesions remains technically challenging. Intravascular ultrasound (IVUS)-guided wiring facilitates safer and more effective procedures. However, aligning IVUS findings with fluoroscopic imaging is challenging due to catheter rotation. We report a novel technique—termed the IDEAL technique—that leverages the deep vein as an anatomical landmark to correct IVUS rotational orientation.

**Case presentation.:**

A 76-year-old female presented with chronic limb-threatening ischemia in the right toe. Contrast-enhanced computed tomography revealed a chronic total occlusion in the right superficial femoral artery. EVT was performed via the right common femoral artery. IVUS revealed that the first guidewire had entered the subintimal space partway through its course. Using preprocedural computed tomography, the deep vein was identified and its clock-face position relative to the artery was determined. This anatomical landmark enabled correction of IVUS image rotation, allowing accurate re-direction of a second guidewire under fluoroscopic guidance, thereby successfully crossing into the intraplaque lumen.

**Conclusions:**

The IDEAL technique quickly provides anatomical orientation by utilizing the deep vein as a landmark during IVUS-guided wiring—making it, quite literally, ideal.

**Supplementary Information:**

The online version contains supplementary material available at 10.1186/s42155-025-00584-5.

## Background

Endovascular therapy (EVT) is widely established as a revascularization strategy for patients with peripheral artery disease (PAD); however, achieving ideal wire crossing can often be technically challenging in clinical practice. The intravascular ultrasound (IVUS)-guided wiring method, which involves redirecting a guidewire into the intraplaque lumen using IVUS imaging, is an effective technique [1] [2]. However, aligning the direction of the center of the artery identified in IVUS images with fluoroscopic images often poses challenges due to the rotational movement of the IVUS catheter. Here, we propose a systematic approach utilizing IVUS, fluoroscopy, and computed tomography (CT) to enhance guidewire redirection accuracy during complex IVUS-guided procedures.

## Case presentation

A 76-year-old female presented with chronic limb-threatening ischemia (CLTI) in the right toe. Her ankle-brachial index was undetectable. Contrast-enhanced CT revealed a chronic total occlusion (CTO) in the right superficial femoral artery (SFA). The length of the CTO was 15 cm, with the total lesion length, including the stenosis, measuring 28 cm.

**Fig. 1:**
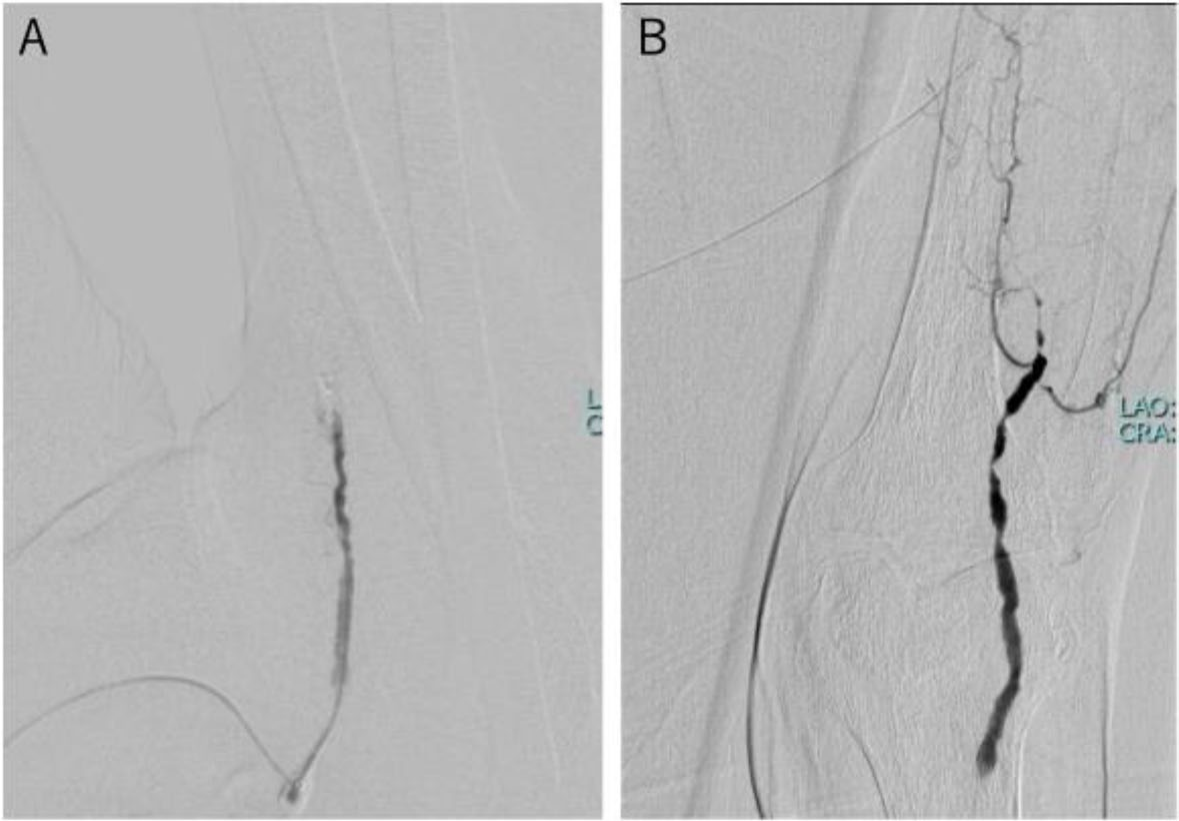
The initial angiography: (**A)** CTO lesion in the SFA, (**B**) Tandem lesions in the popliteal artery and CTO in the below-the-knee arteries

**Fig. 2:**
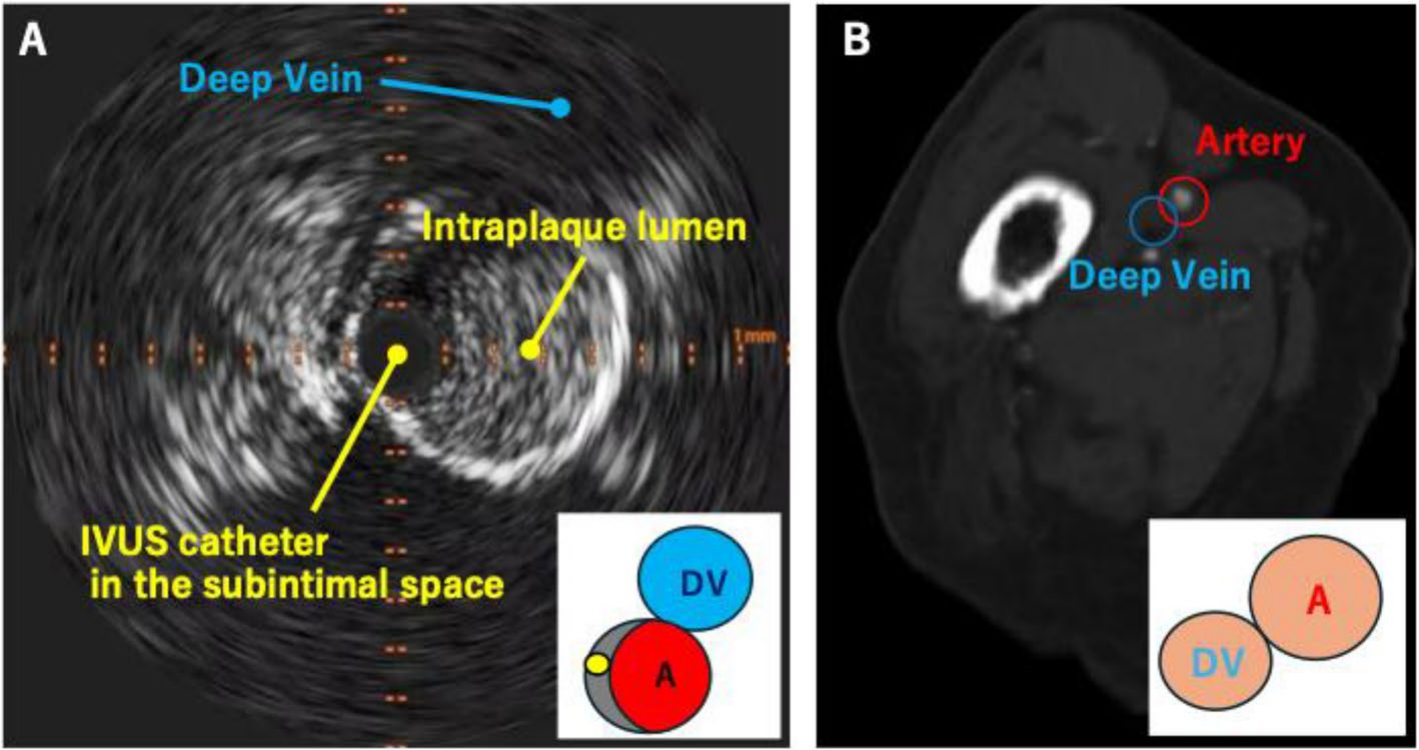
**(A)** IVUS image; The deep vein is located at the 1 o’clock position relative to the artery on the clock-face orientation. (**B**) CT image; The deep vein is located at the 8 o’clock position relative to the artery on the clock-face orientation. Abbreviations: DV, deep vein; A, artery

A 5-Fr guiding sheath was inserted into the right common femoral artery via an ipsilateral antegrade approach. Initial angiography demonstrated CTO of the SFA, tandem lesions in the popliteal artery, and the below-the-knee arteries (Fig. 1AB). A 0.014-inch guidewire (Gladius; Asahi Intecc, Aichi, Japan) and an IVUS catheter (Eagle Eye Platinum short-tip; Philips Amsterdam, Netherlands) appeared to have advanced into the subintimal space of the SFA CTO. We identified the deep vein on the IVUS image and confirmed its position relative to the artery using clock-face orientation (Fig. 2A). Preprocedural CT images at the corresponding anatomical level were then reviewed to verify the course of the deep vein in relation to the SFA, also using clock-face orientation (Fig. 2B).

**Fig. 3:**
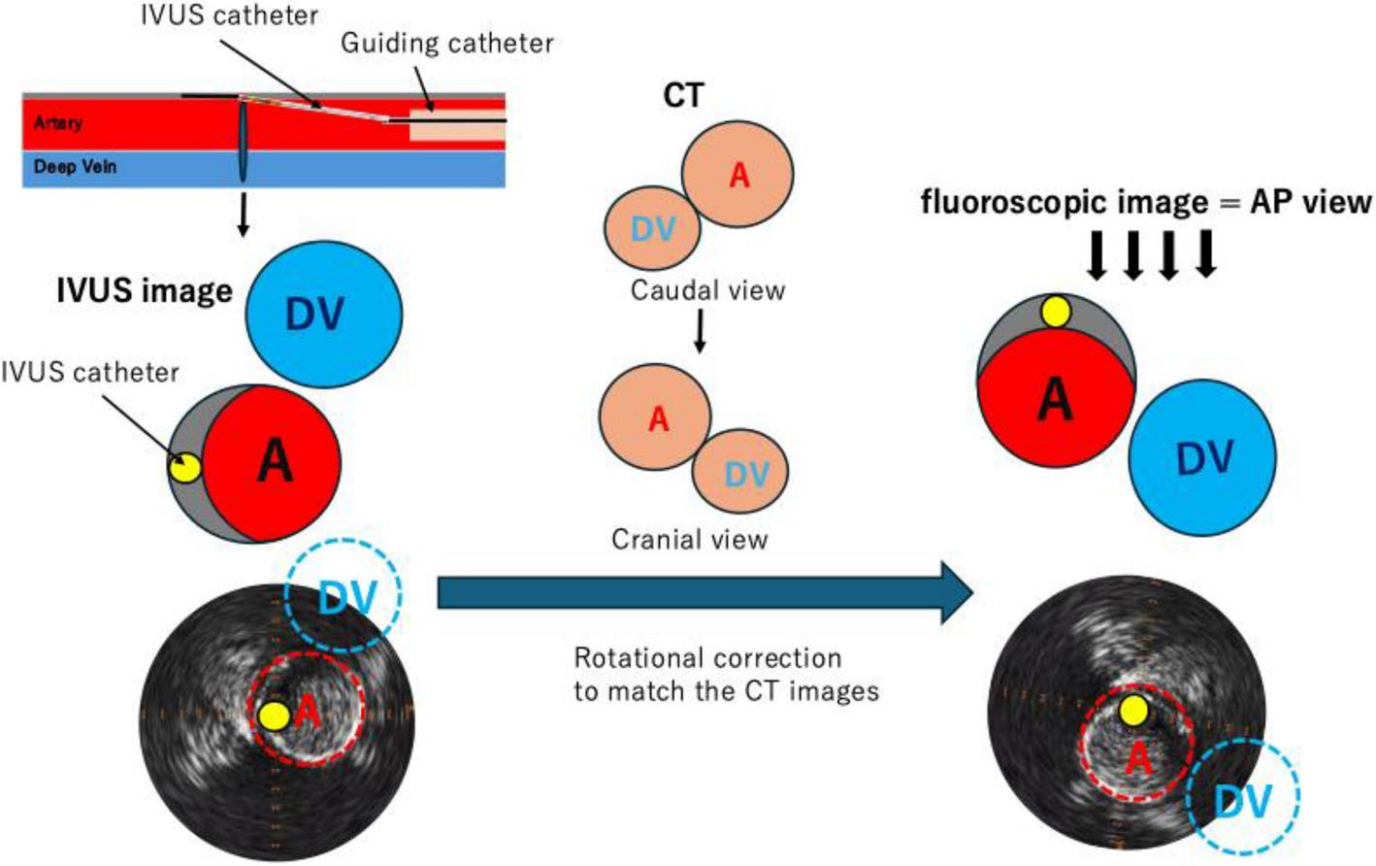
Schematic illustration of the alignment process between IVUS and CT images. The IVUS image demonstrated the relative positions of the artery and deep vein. Since the CT images were originally displayed in a caudal view, they were converted to a cranial view to match the IVUS orientation. Rotational correction of the IVUS image was performed using the deep vein as a landmark. As a result of this correction, it was determined that the IVUS catheter was positioned at the 12 o’clock direction, and the center of the artery was located at the 6 o’clock direction relative to the catheter. Abbreviations: DV, deep vein; A, artery; AP, anteroposterior; CT, computed tomography

To establish precise anatomical orientation, IVUS images were aligned with CT data. As the CT images were originally displayed in a caudal view, they were converted to a cranial view　to match the IVUS images. Rotational adjustment of the IVUS images was performed using the deep vein as a landmark. As a result of this adjustment, the 12 o'clock to 6 o'clock axis corresponded to the vertical axis, aligning with the anteroposterior view on fluoroscopic imaging. Subsequently, it was confirmed that the IVUS catheter was positioned at the 12 o'clock direction and that the center of the artery was located at the 6 o'clock direction relative to the IVUS catheter (Fig. 3).

**Fig. 4:**
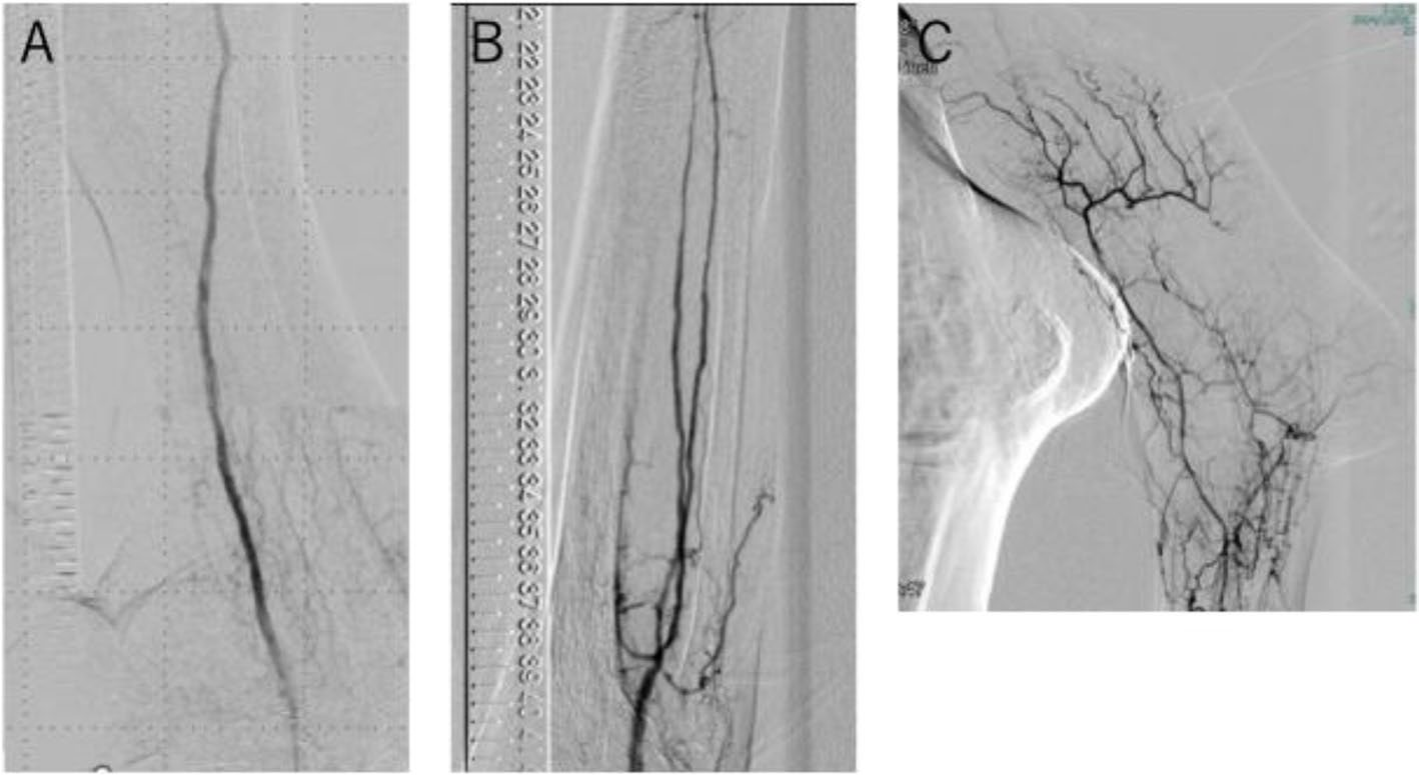
The final angiography: (**A**) superficial femoral artery, (**B**) Below-the-knee arteries, (**C**) Below-the-ankle arteries

Under fluoroscopic guidance, a 0.014-inch CTO guidewire (Astato XS 9–12; Asahi Intecc, Aichi, Japan), used as the second wire, was initially advanced toward the right side of the IVUS catheter (3 o’clock direction). By subsequently torquing the wire 90 degrees clockwise, it was successfully redirected toward the 6 o’clock direction, thereby achieving entry into the intraplaque lumen (Supplementary Video). We performed predilatation using a 5.0-mm balloon (JADE; OrbusNeich, Hong Kong, China), which resulted in satisfactory luminal expansion without significant intimal dissection. EVT was also performed for the below-the-knee lesion. Final dilatation of the SFA was achieved using a drug-coating balloon (Ranger; Boston Scientific, Marlborough, MA, USA). Final angiography demonstrated good in-line flow to the wound (Fig. 4A-C). The guidewire took 15 min to traverse the CTO, successfully passing through the intraplaque lumen along its entire length. The total procedure time, including the below-the-knee lesion, was 125 min, the radiation exposure time was 47 min, and 85 mL of contrast agent was used.

## Discussions

IVUS-guided wiring has been shown to improve both the safety and long-term outcomes of EVT for PAD [3] [4], particularly when the guidewire can be successfully advanced through the intraplaque route. As previous reports have demonstrated, intraplaque wiring under IVUS guidance enables safer and more effective balloon angioplasty, reducing the risk of vessel dissection and restenosis [5].

Furthermore, IVUS guidance facilitates precise assessment of vessel architecture, including accurate measurement of vessel diameter and lesion length, allowing for the selection of appropriately sized balloons and devices. This optimization contributes to improved long-term patency, reduces the likelihood of reintervention, and ultimately lowers healthcare costs [6]. Especially for CTO lesions, IVUS-guided wiring should be adopted to ensure that the guidewire passes through the entire length of the CTO via the intraplaque route.

Several methods for achieving intraplaque tracking under IVUS guidance have been described in previous reports [1] [2], including the tip detection technique widely used in coronary interventions [7]. However, these approaches typically require specific IVUS systems equipped with asymmetrical transducer structures, which allow for visualization of the IVUS catheter’s position within the vessel wall and planning of second guidewire advancement based on fluoroscopic landmarks. A limitation of such IVUS systems is that they constrain the procedure to particular devices and do not support the IVUS-preceding approach we previously reported, in which the IVUS catheter is advanced ahead of the guidewire [8].

The method described in the present case overcomes these limitations by utilizing the deep vein as a fixed anatomical landmark. By identifying the relative clock-face position of the deep vein to the artery using preprocedural CT and correlating it with IVUS images, the precise rotational orientation of the IVUS catheter can be determined regardless of the system used. This enables the operator to intuitively understand the directional relationship between the IVUS image and the fluoroscopic view, allowing for accurate advancement of a second guidewire toward the intraplaque lumen.

This technique consists of three steps and is relatively simple. In the case of an antegrade approach, the first step is to identify the deep vein on the IVUS image at the site where the first guidewire has entered the subintimal space. The next step is to horizontally flip the CT image and accurately position the deep vein to anatomically align with the IVUS image. To determine the CT image that corresponds to the horizontal level of the cross-sectional image observed on IVUS, adjacent bony landmarks, an external radiopaque ruler placed within the fluoroscopic field of view, and scout images from the CT series are useful. The final step is to reinterpret the orientation into the fluoroscopic anteroposterior view and advance the second guidewire toward the center of the vessel. The direction of the wire tip under fluoroscopy can be approximately estimated in increments of 45 degrees. In contrast to coronary interventions, peripheral arteries have larger vessel diameters and broader target intraplaque lumens, allowing for sufficiently practical wire manipulation. It is possible to easily identify the direction of the intraplaque lumen relative to the first guidewire using only the anteroposterior view. However, if necessary, rotational angiography can be used in conjunction with the established direction.

For a retrograde approach, the first step remains the same, but in the second step, flipping the CT image is unnecessary, as the IVUS image is already in the caudal view and corresponds with the CT. The IVUS image can simply be rotated to match the position of the deep vein, allowing intuitive determination of wire direction without additional image processing. This applies, for example, to retrograde IVUS cases such as treatment of the femoro-popliteal region via a trans-ankle approach or the aorto-iliac region via a trans-femoral approach. In the third step, the orientation obtained from the IVUS image is incorporated into the fluoroscopic anteroposterior view and wire manipulation, where it is preferable for the cranial side to be displayed at the top of the monitor.

The anatomical relationship between arteries and their associated deep veins on CT appears to be generally consistent, with low interindividual variability. We retrospectively analyzed lower limb CT angiography in 100 consecutive patients who underwent EVT at our institution from May 2025 backward. In the typical right femoropopliteal region, the deep vein was located at the 3 to 4 o’clock position relative to the superficial femoral artery at the femoral head level. As it progressed distally, the deep vein rotated clockwise and shifted to the 5 to 6 o’clock position at the lesser trochanter level and to the 7 to 8 o’clock position from the distal lesser trochanter to the popliteal artery P1 segment. From P1 to P2, the deep vein rotated counterclockwise to the 6 o’clock position and moved to approximately 5 o’clock by the P3 level. A mirror-image pattern was observed on the left side. When this trajectory was defined as the"normal"pattern, the proportion of patients in whom the deep vein deviated from this course by less than 20% of the total segment length was 90% on the right side and 88% on the left side. Therefore, this technique may be applicable even in the absence of preprocedural CT. Leveraging this anatomical consistency may further enhance the technique’s versatility in clinical practice. Inserting a guidewire into a vein or performing venography to visualize the deep vein on fluoroscopic images may allow for more accurate fusion of the IVUS image with the fluoroscopic image. However, the course of the deep vein is typically identifiable on preprocedural CT, and individual variation in its anatomical relationship is minimal. Therefore, visualization of the deep vein is generally unnecessary in most cases.

The tip detection technique is also useful for directing the guidewire toward the intended direction, but it differs from the IDEAL technique in that it requires multiple torque attempts within the subintimal space, which can prolong the procedure and increase the risk of enlarging the subintimal space. In contrast, the IDEAL technique determines direction based on IVUS and fluoroscopic images alone, without the need for torquing trials, minimizing these risks. However, IDEAL does not allow precise confirmation of the guidewire's rotation angle. By combining IDEAL with the tip detection technique, finer adjustments to the wire tip can be made, leveraging the strengths of both approaches and improving procedural success.

In cases of long CTO lesions, the direction of the intraplaque lumen is reassessed using the IDEAL technique each time the first guidewire enters the subintimal space. The second guidewire can then be directed into the intraplaque lumen, and both the IVUS catheter and the guiding catheter can be advanced along it. Once the direction is established, even high-penetration guidewires with heavy tip loads can be used with relative safety. By repeating this process, this technique can be applied even to long CTO lesions.

There are, however, several limitations to this approach. First, in cases with heavy arterial calcification, acoustic shadowing may render the deep vein unvisualizable on IVUS at certain levels. However, in many cases, the deep vein can still be visualized in adjacent segments proximal or distal to the calcification, which is sufficient to aid in estimating directional orientation. In some cases, the calcification itself, which is visible on fluoroscopy, may serve as an alternative reference marker. Second, in the iliac artery region, the deep vein may be located too far from the artery to function as a reliable landmark. Finally, while deep veins in below-the-knee lesions are generally visible on IVUS, their anatomical correlation with arteries on CT can be less distinct, complicating directional orientation. These anatomical characteristics can pose limitations on the applicability of the IDEAL technique in such regions. Nevertheless, in cases where the anatomical conditions are favorable, the technique can be extended to these non-femoro-popliteal regions.

## Conclusion

The IDEAL technique quickly provides anatomical orientation by utilizing the deep vein as a landmark during IVUS-guided wiring—making it, quite literally, ideal. Given our accumulating evidence, including the present case, if we confirm a deep vein running parallel to the target artery on IVUS, we highly recommend this technique.

## Supplementary Information

Supplementary video. This video provides a comprehensive explanation of the entire IDEAL technique in the case of an antegrade approach.

## Data Availability

The data and material on the case report are available from the corresponding. author, MS, upon reasonable request.

## Abbreviations

IDEAL: IVUS-guide wiring using the deep vein as a landmark
EVT: Endovascular treatment
CTO: Chronic total occlusion
PAD: Peripheral artery disease
IVUS: Intravascular ultrasound
SFA: Superficial femoral artery
CLTI: Chronic limb-threatening ischemia
CT: Computed tomography
